# Unremodeled GPI-anchored proteins at the plasma membrane trigger aberrant endocytosis

**DOI:** 10.26508/lsa.202402941

**Published:** 2024-11-22

**Authors:** Li Chen, David K Banfield

**Affiliations:** Division of Life Science, The Hong Kong University of Science and Technology , Kowloon, SAR of China

## Abstract

The plasma membrane has a complex organization that includes the polarized distribution of membrane proteins and lipids. Glycosylphosphatidylinositol-anchored proteins (GPI-APs) are ubiquitously expressed in eukaryotes and represent a functionally diverse, extensively remodeled, ER-derived group of proteins critical for the organization and function of the plasma membrane. Little is known about how the transport of incompletely remodeled GPI-APs to the plasma membrane affects cell function. Here, we investigated how failure to remodel mannose 2 (Man2) of the GPI moiety impacted endocytic activity on the plasma membrane. We find that Man2 unremodeled GPI-APs increased membrane disorder and generated a stress response that triggered abnormal ubiquitin- and clathrin-dependent endocytosis. The resulting stress-induced endocytosis disrupted the trafficking repertoire of a subset of plasma membrane proteins, which were redirected, via the multivesicular body, to numerous small vacuoles for degradation. Our findings highlight the critical importance of GPI-AP Man2 remodeling for maintaining the integrity and homeostasis of the plasma membrane.

## Abstract (precis)

A unique plasma membrane stressor induces vacuolar degradation of abnormally endocytosed proteins.

## Introduction

The plasma membrane serves as a semipermeable barrier between the extracellular and intracellular environments of the cell. Much is known about the role of cytoskeletal components, membrane proteins, and lipids in establishing a dynamically responsive plasma membrane. Moreover, it is well understood that the interaction between certain proteins and lipids can lead to the formation of domains in the plasma membrane that are critical for the generation of signaling platforms (Simons & Toomre, 2000; Laude & Prior, 2004). Signaling platforms allow cells to respond to certain stimuli, including alterations in osmotic pressure, external pH, and the effects of cellular aging (Simons & Toomre, 2000; Parton & Hancock, 2004; Galluzzi et al, 2018). In some instances, the response to a particular stress signal can elicit a transient arrest in cell growth (Jiménez et al, 2020; Tognetti et al, 2020; de Nadal & Posas, 2022).

The extent to which aberrant proteins might impinge on plasma membrane homeostasis is difficult to assess as misfolded proteins activate stress responses and protein degradation soon after their synthesis via ER-associated degradation, the unfolded protein response, or endosome–Golgi-associated degradation (Benyair et al, 2022; Krshnan et al, 2022; Wiseman et al, 2022; Bhaduri et al, 2023). In contrast, misfolded glycosylphosphatidylinositol-anchored proteins (GPI-APs), which are ultimately sorted to the plasma membrane, are poor substrates for ER-based degradation pathways. In yeast cells, misfolded GPI-APs are redirected to the vacuole for degradation (Sikorska et al, 2016; Lemus et al, 2021; Lemus & Goder, 2022), whereas in mammalian cells, they are robustly retrieved from the plasma membrane (Satpute-Krishan et al, 2014). To date, little is known about the post-ER fate of GPI-APs in which the protein is folded correctly, but the GPI moiety is incorrectly remodeled including: whether they can be sorted to the plasma membrane, and if so the impact such proteins might have on plasma membrane homeostasis (Chen et al, 2021).

GPI-APs are an evolutionarily conserved class of eukaryotic membrane proteins assembled in the ER via the en bloc addition of a lipid moiety after the removal of a signal sequence (Muñiz & Zurzolo, 2014; Paladino et al, 2015; Kinoshita & Fujita, 2016; Kinoshita, 2020). The GPI anchor of nascent GPI-APs undergoes extensive remodeling before the transport of these proteins from the ER to the plasma membrane via the Golgi (Muñiz & Zurzolo, 2014; Kinoshita, 2020). Although GPI-APs are ubiquitous in eukaryotic cells, their functions can vary tremendously and include roles in cell–cell adhesion, cell wall biosynthesis, signal transduction, the immune response, and the organization of lipids in the plasma membrane, and as enzymes and receptors (Simons & Toomre, 2000; Laude & Prior, 2004; Paladino et al, 2015).

The GPI anchor is comprised of a glycan core that consists of three mannose residues, one of which is attached to the protein (Man 3) and the other two are modified through the attachment of phosphoethanolamine (EtNP). Gpi7p (PIG-V in human) transfers EtNP to Man2 of GPI-APs in the ER (Benachour et al, 1999). Ted1p (PGAP5 in human) in the ER and Dcr2p in the Golgi remove the EtNP on Man2 before the GPI-APs reach the PM (Fujita et al, 2009; Manzano-Lopez et al, 2015; Chen et al, 2021). The removal of EtNP from Man2 is an evolutionarily conserved remodeling event, whereas the removal of EtNP from Man1 has not yet been observed on human GPI-APs (Kinoshita, 2020). Glucosamine on Man1 is linked to phosphatidylinositol (PI), which is in turn modified by the addition and remodeling of various lipids (Kinoshita & Fujita, 2016; Kinoshita, 2020). Most GPI anchor remodeling events occur in the ER, and they are often a prerequisite for the robust export of GPI-APs from this organelle (Manzano-Lopez et al, 2015; Rodriguez-Gallardo et al, 2022). In metazoans, some GPI-APs are cleaved and thereafter are released from the extra-cytoplasmic side of the plasma membrane (Fujihara & Ikawa, 2016; Müller, 2018; Müller & Müller, 2023), whereas others, such as the folate receptor, are endocytosed. In contrast, numerous GPI-APs in budding yeast cells are cleaved once they reach the cell surface, whereupon the protein becomes a constituent of the cell wall (Kitagaki et al, 2002; Yin et al, 2007; Vogt et al, 2020). Relatively little is understood about features of the GPI moiety that are required for their cleavage from the protein, nor is it understood how remodeling defects impact the function of GPI-APs that reach the plasma membrane (Müller, 2018). Nevertheless, GPI-AP remodeling defects and/or deficiencies in the cleavage of the GPI moiety from the proteins are likely to impact plasma membrane homeostasis and have far-reaching physiological consequences (Paladino et al, 2015; Sevcsik et al, 2015; Fujihara & Ikawa, 2016).

We have previously reported that incompletely remodeled GPI-APs elicit stress responses that include activation of the cell wall integrity pathway and non-canonical activation of the spindle assembly checkpoint (SAC) (Chen et al, 2021). Here, we report that a failure to remove EtNP from Man2 of yeast GPI-APs renders them poor substrates for cleavage. Moreover, we show that at least one unremodeled, uncleaved GPI-AP is not endocytosed. Rather, the presence of incompletely remodeled GPI-APs in the plasma membrane increased the membrane disordered phase of membranes and triggered abnormal ubiquitin- and clathrin-dependent endocytosis of certain proteins, which were thereafter degraded in an ESCRT-dependent manner in the vacuole.

We conclude that Man2 unremodeled GPI-APs disrupt plasma membrane homeostasis and trigger aberrant endocytosis. Our findings highlight the critical importance of GPI-AP Man2 remodeling for maintaining the integrity and homeostasis of the plasma membrane. The identification of abnormal clathrin-mediated endocytosis (CME) as a response to such perturbations suggests a novel means by which plasma membrane stress signals are transmitted to the interior of the cell.

## Results

### Man2 unremodeled GPI-APs have altered biochemical properties and induce increased membrane disorder in cells

Our previous study (Chen et al, 2021) demonstrated that GPI-APs bearing EtNP on Man2 could still be delivered to the plasma membrane, and that their presence there induced a stress signal that triggered non-canonical activation of the SAC. From these findings, we hypothesized that GPI-APs containing EtNP on Man2 disrupt plasma membrane homeostasis in some manner that generates a stress response and concomitant arrest of cell growth.

To address the nature of the prospective cellular perturbation, we initially considered the possibility that Man2 unremodeled GPI-APs might display altered biochemical properties, which impact the functional integrity of the plasma membrane. A commonly used method to examine the characteristics of membrane proteins is to monitor their partitioning in detergent extracts (Lingwood & Simons, 2007). When detergent extraction is conducted at 4°C, certain integral membrane proteins including GPI-APs are found predominantly in detergent-resistant membranes (DRMs) (Bagnat et al, 2000, 2001).

**Figure 1.**
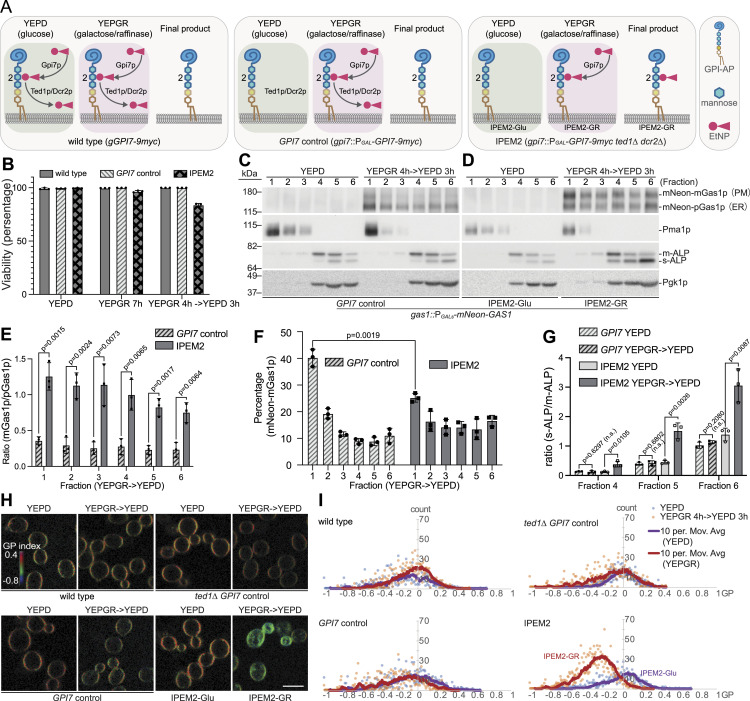
Man2 unremodeled GPI-APs display altered biochemical properties and increase the lipid disordered phase of membranes. **(A)** Schematic representations and genotype annotations of the commonly used yeast strains in this study. **(B)** ∼80% of IPEM2-GR cells remain viable 7 h after induction of Gpi7p synthesis. 100 cells from each biological repeat (n = 3) were scored, and the average number of viable cells plus the SD are presented. **(C)** Partitioning profile of proteins from GPI7 control cells into detergent-resistant membranes. See panel (A) for strain details. **(D)** Partitioning profile of proteins from IPEM2-Glu and IPEM2-GR cells into detergent-resistant membranes. See panel (A) for strain details. **(C, D)** depict one replicate of three independent replicates. Pma1p is a protein that displays resistance to extraction with detergent, whereas ALP (Pho8p) and Pgk1p are vacuolar and cytoplasmic proteins, respectively, that are reportedly not resistant to extraction with detergent. **(C, D, E)** Quantification of the ratio between mNeon-mGas1p versus mNeon-pGas1p for each fraction from experiments depicted in (C, D) (average + SD). **(C, D, F)** Quantification of the percentage of mNeon-mGas1p from each fraction from experiments depicted in panels (C, D). **(C, D, G)** Quantification of the ratio between s-ALP versus m-ALP in fractions 4–6 in panels (C, D). Three independent detergent extraction datasets from cells grown in YEPGR media were used in the calculations, and the average value plus the SD are presented. **(H)** GP values from the indicated di-4-ANEPPDHQ-stained yeast strains. The represented GP images are pseudo-colored and extend over the range indicated by the inserted color bar. **(E, I)** Corresponding GP value histogram of cells presented in (E) (n = 40).

To investigate the properties of Man2 unremodeled GPI-APs in detergent extraction experiments, we used a yeast strain in which EtNP was permanently added to Man2, hereafter referred to as the IPEM2 (induced permanent phosphoethanolamine on mannose 2; Chen & Banfield, 2022) strain. IPEM2 cells can grow in the presence of glucose (conditions hereafter denoted as IPEM2-Glu) as the expression of *GPI7* (which encodes the sole enzyme that adds EtNP to Man2 in budding yeast cells) is suppressed. However, when IPEM2 cells are grown in galactose/raffinose-containing media (conditions hereafter denoted as IPEM2-GR), EtNP is added to Man2 but cannot be removed as this strain lacks the genes encoding the enzymes that remove EtNP from Man2 of GPI-APs (i.e., *TED1* and *DCR2*; Chen et al, 2021; Chen & Banfield, 2022). Fig 1A depicts the commonly used strains in this study and their corresponding genotypes. Importantly, although permanent attachment of EtNP to Man2 arrests cell growth, ∼80% of IPEM2-GR cells remained viable over the 7-h assay period used for the experiments we describe in this study (Fig 1A and B).

**Figure S1.**
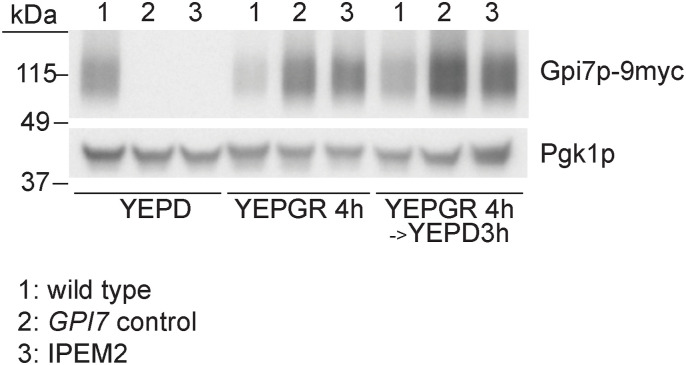
Expression profile of *GPI7* in IPEM2-GR cells and control strains. *GPI7* was expressed as a C-terminal fusion protein to nine copies of the MYC epitope using either the *GPI7* promoter or the *GAL 1/10* promoter, as indicated. WCEs from yeast strains grown under the conditions indicated were subject to SDS–PAGE and immunoblotting. Pgk1p serves as a gel loading control.

To monitor the trafficking of GPI-APs, we used Gas1p as this yeast GPI-AP has been extensively studied (Nuoffer et al, 1991; Ragni et al, 2007). Gas1p was expressed in the IPEM2 strain as an attenuated GAL1/10 promoter–driven (galactose-inducible/glucose-repressible) fusion to mNeon-Green (mNeon-Gas1p), where the coding sequence of mNeon-Green was inserted into the *GAS1* gene after the sequence that encodes the signal peptide (Chen et al, 2021; Chen & Banfield, 2022). Because of the attenuated GAL1/10 promoter, the induction of mNeon-Gas1p occurs later than that of Gpi7p (which is driven by a WT GAL1/10 promoter), ensuring that mNeon-Gas1p has EtNP attached to Man2 (Chen & Banfield, 2022). The molecular weight of ER-resident Gas1p (pGas1p) differs from its plasma membrane–localized form (mGas1p) because of differences in their glycosylation profiles (Nuoffer et al, 1991). The trafficking of mNeon-Gas1p and expression of *GPI7* were monitored by immunoblotting (Figs 1C and D and S1).

As anticipated, in cells expressing the Man2 remodelases Ted1p and Dcr2p (i.e., the GPI7 control cells, Fig 1C) (Chen et al, 2021), the mature/fully remodeled form of mNeon-Gas1p (mGas1p) was found predominately in DRM fractions as judged by the cofractionation with the canonical yeast DRM-resident protein Pma1p (Fig 1C). In IPEM2-GR cells, however, we observed an incremental increase in the amount of the mature form of mNeon-mGas1p across all fractions. This observation may indicate that more mGas1p is associated with the plasma membrane in IPEM2-GR cells, whereas more mGas1p is cleaved in the control strains (Fig 1D and E). As Gas1p is reportedly a cell wall–associated protein (Yin et al, 2005, 2007), unremodeled Gas1p may be a poor substrate for cleavage and linkage to cell wall glycans.

In the *GPI7* control, ∼40% of total mNeon-mGas1p was found in fraction 1 (Fig 1C and F), whereas in IPEM2-GR cells, this was reduced to ∼25% of the mNeon-mGas1p (Fig 1F). Nonetheless, the partitioning of Pma1p, Pho8p, and Pgk1p (Fig 1D) was similar across all strains and growth conditions tested although there was an overall reduction in the amount of Pma1p IPEM2-GR cells (Fig 1C and D). The reduction in Pma1p may indicate an underlying defect in the sorting of this protein to the plasma membrane in IPEM2-GR cells (Bagnat et al, 2001). We concluded that Gas1p containing EtNP on Man2 displayed altered biochemical characteristics that distinguished it from the fully remodeled form of the protein.

The findings presented in Fig 1B–G prompted us to explore whether Man2 unremodeled GPI-APs affected membrane lipid homeostasis. The plasma membrane is comprised of lipid domains that display either a more ordered state (L_O_), enriched in sterols and sphingolipids, or a more disordered state (L_D_), enriched in lipids containing unsaturated fatty acids and exhibiting greater mobility of lipids (Sezgin et al, 2017). Maintaining the balance between L_O_ versus L_D_ domains is thought to play a central role in establishing the functional landscape of the plasma membrane (Simons & Toomre, 2000; Laude & Prior, 2004). To address the prospect that GPI-APs in IPEM2-GR cells might perturb the ratio of L_O_ to L_D_ domains, we employed the aminonaphthylethenylpyridinium voltage-sensitive dye di-4-ANEPPDHQ in quantitative confocal fluorescence microscopy experiments (Owen et al, 2011). This dye is excited at 488 nm yet results in a peak emission wavelength of ∼560 nm in the lipid ordered phase and ∼620 nm in the disordered phase. The spectral shift of di-4-ANEPPDHQ to ∼560 nm or ∼620 nm allows calculation of the generalized polarization (GP), which is a relative but quantitative measure of lipid packing (Jin et al, 2005; Zhao et al, 2015; Amaro et al, 2017). GP values range from −1 to +1. A lower GP value, relative to controls, indicates a higher percentage of L_D_ and vice versa for L_O_. By measuring the fluorescence intensity of the two emissions, we were able to monitor the changes in L_O_/L_D_. These experiments revealed an overall increase in the L_D_ proportion of membranes (or relatively loosely packed membranes) in IPEM2-GR cells relative to IPEM2-Glu cells and GPI7 control cells (Fig 1H and I). Thus, the biochemical properties of GPI-APs in IPEM2-GR cells are altered, and these cells display an increase in membrane L_D_.

### Gas1p is not endocytosed in IPEM2-GR cells

**Figure 2.**
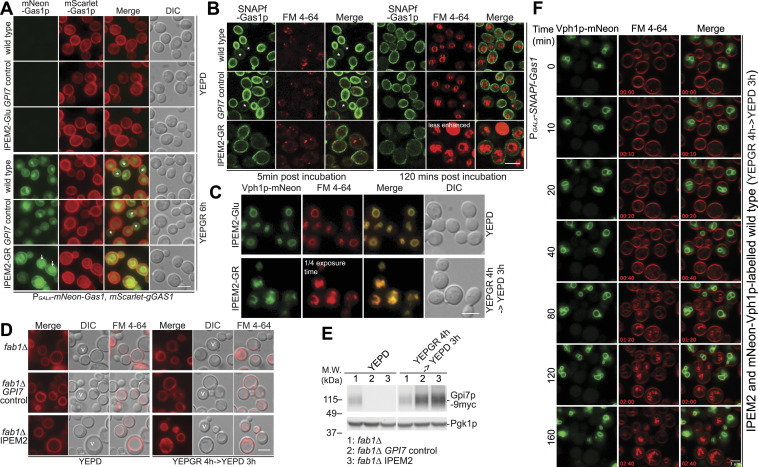
GPI-AP Gas1p is not endocytosed in IPEM2-GR cells. **(A)** Newly synthesized Gas1p is targeted to the nascent bud in WT and *GPI7* control cells, but uniformly distributed on the plasma membrane in IPEM-GR cells. The asterisks indicate the location of newly synthesized mNeon-Gas1p in WT and *GPI7* cells, whereas arrows indicate the ER. The scale bar is 5 μm. **(B)** mNeon-Gas1p from IPEM2-GR cells reaches the plasma membrane but is not endocytosed. The indicated strains were labeled with SNAP-Surface 488, and the fate of the labeled protein was assessed 5 and 120 min after the removal of excess dyes and galactose + raffinose. The asterisks indicate the exclusion of de novo–synthesized plasma membrane–localized SNAPf-Gas1p from the mother cell in WT and *GPI7* control cells. The scale bar is 5 μm. **(C)** Vacuole-resident integral membrane protein Vph1p-mNeon localizes to FM 4-64–positive membrane structures in IPEM2-GR cells. The scale bar is 5 μm. **(C, D)** Membranous structures that accumulate in IPEM2-GR cells (in panels (C, D)) represent numerous small vacuoles. Note that deletion of *FAB1* in IPEM2-Glu and IPEM2-GR cells generates a single large vacuole. The scale bar is 5 μm. V denotes the vacuole. **(E)** Expression profile of Gpi7p in the yeast strains depicted in (E). Pgk1p serves as a gel loading control. **(F)** Kinetics of FM 4-64 uptake and transport are indistinguishable in IPEM2-GR and WT cells. WT cells expressing Vph1p-mNeon were mixed with IPEM2-GR cells (as indicated), and thereafter, cells were labeled with an FM 4-64 dye. Cells were imaged over the time course shown. Note that only IPEM2-GR cells show increased FM 4-64 fluorescence intensity (from the 80-min time point onward). The scale bar is 3 μm.

In budding yeast cells, many GPI-APs are constituents of the cell wall, where the GPI moiety is cleaved at the plasma membrane and the protein is then covalently attached to the cell wall (Yin et al, 2005; Müller, 2018). The results of our spectral ratiometric imaging experiments (Fig 1H and I) revealed that membranes from IPEM2-GR cells exhibited an increase in L_D_, which likely impacts the extent to which regions of the plasma membrane cluster sterols and sphingolipids (Simons & Toomre, 2000; Laude & Prior, 2004). As Gas1p appeared to be delivered to the cell surface (Chen et al, 2021), the observed membrane perturbations in IPEM2-GR cells may be a consequence of the presence of Man2 unremodeled Gas1p (and GPI-APs more generally) in the plasma membrane. To investigate this further, we examined the fate of newly synthesized Gas1p in IPEM2-GR cells. We generated an IPEM2 strain constitutively expressing mScarlet-Gas1p and a copy of mNeon-Gas1p whose expression was under the control of the attenuated GAL1/10 promoter (Fig 2A). In control strains, de novo–synthesized mNeon-Gas1p (YEPGR, 6 h) was predominantly localized to the incipient daughter cell (asterisk, Fig 2A). Daughter cells are easily distinguished from the mother cell as budding yeast cells grow anisotropically, and therefore, during earlier stages of the cell division cycle, daughter cells appear smaller in size than their mother. However, in IPEM2-GR cells, both mNeon-Gas1p and mScarlet-Gas1p were uniformly distributed around the cell periphery and to ER membranes (arrows in Fig 2A). The presence of mNeon-Gas1p in the ER is expected as IPEM2-GR cells lack *TED1* and will therefore exhibit a delay in the export of Gas1p from the ER (Manzano-Lopez et al, 2015). Taken together with the data from the DRM experiments (Fig 1D), these findings suggest that in IPEM2-GR cells, Gas1p with EtNP on Man2 may be a poor substrate for cleavage and cross-linking to the cell wall and therefore free to diffuse in the plasma membrane (Fig 2A). Indeed, failure to remove EtNP from Man1 of GPI-APs has been shown to impair cleavage and cross-linking of two GPI-APs in budding yeast cells (Vazquez et al, 2014).

**Figure S2.**
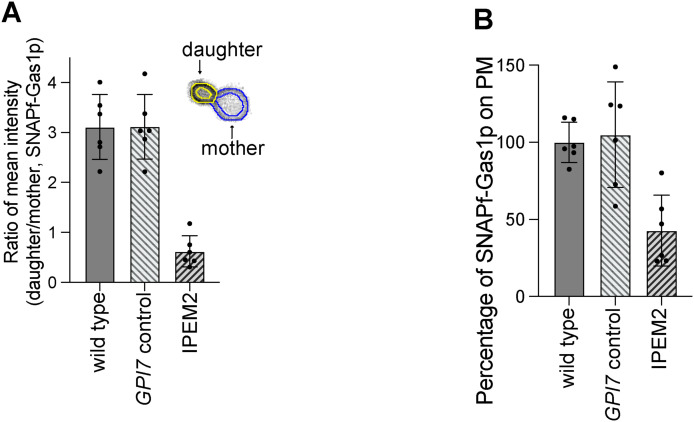
Quantification of SNAPf-Gas1p for Fig 2B. **(A)** Distribution of SNAPf-Gas1p in IPEM2-GR mother and daughter cells. **(B)** Relative percentage of SNAPf-Gas1p on the plasma membrane of IPEM2-GR cells. n = 6.

Our speculation that Gas1p may not be cleaved prompted us to ask whether plasma membrane–resident Gas1p might be subject to endocytosis in IPEM2-GR cells, as this could provide a means to re-establish plasma membrane homeostasis more generally if applied to additional GPI-APs (Fig 1H and I). To monitor the fate of plasma membrane–localized Gas1p in IPEM2-GR cells, we introduced a GAL1/10-inducible SNAP-tagged Gas1p fusion protein (SNAPf-Gas1p) and used the membrane-impermeable dye SNAP-Surface 488 to label only SNAPf-Gas1p fusion proteins that had reached the plasma membrane (Keppler et al, 2004) (Fig 2B). Although FM 4-64–positive puncta (presumably endosomes) were evident in all strains examined after 5 min of incubation at 25°C, and after 120 min, the limiting membrane of the vacuole was also labeled (Fig 2B). After 120 min at 25°C, neither the controls nor IPEM2-GR cells showed any evidence of internalized SNAPf-Gas1p (Fig 2B). In IPEM2-GR cells, de novo–synthesized SNAPf-Gas1p (5-min time point) was distributed equally on the surface of mother and daughter cells (Figs 2B and S2A), which is consistent with the data obtained for IPEM2-GR cells presented in Fig 2A and adds further support to our hypothesis that IPEM2-GR cells are deficient in cleaving Gas1p. In contrast, the distribution of SNAPf-Gas1p in WT and *GPI7* control cells showed an asymmetric distribution whereby the daughter cell, but not the mother cell, was predominantly labeled (Fig 2B, 5-min post-incubation; Fig S2A). Fluorescence quantification also revealed that IPEM2-GR cells display ∼40% of levels of SNAPf-Gas1p on their surface as WT and *GPI7* control cells (Fig S2B).

In addition, in IPEM2-GR cells FM 4-64 accumulated in brightly fluorescent internal structures that we surmised represented small (and often clustered) vacuoles (Fig 2B and Video 1). As all strains were treated identically, the intensity of internal FM 4-64 fluorescence in IPEM2-GR cells suggests that these cells have physiochemically abnormal vacuoles that increase the fluorescence intensity of the dye.

Video 1 IPEM2-GR cells contain fragmented vacuoles. Cells were labeled with FM 4–64 and imaged by confocal microscopy. Z-stack images of the vacuoles are shown in the Z-plane starting from the top. Download video

To confirm the vacuolar origin of the FM 4-64–positive internal structures in IPEM2-GR cells, we introduced an mNeon-tagged integral membrane protein that localizes to the limiting membrane of the vacuole (Vph1p-mNeon; Fig 2C). As anticipated, Vph1p-mNeon colocalized with FM 4-64, suggesting that these structures are of vacuolar origin. To address whether IPEM2-GR cells contain clusters of small vacuoles, we deleted *FAB1*. *FAB1* encodes the only 1-phosphatidylinositol-3-phosphate 5-kinase in the yeast genome, and Fab1p is responsible for the synthesis of PI3,5P2 (Yamamoto et al, 1995). Importantly, cells that lack *FAB1* are defective in vacuolar fission and consequently contain a single large vacuole (Yamamoto et al, 1995). When we examined vacuole morphology in IPEM2-GR *fab1*Δ cells stained with FM 4-64, we observed a single large vacuole (Fig 2D and E). These data clearly indicate that a large vacuole forms in IPEM2-GR cells lacking *FAB1*, supporting our hypothesis that the numerous small intracellular membranous structures are indeed fusogenic vacuoles. Note that the single large vacuole seen with DIC is coincident with structures that are labeled with FM 4-64 when images are merged. Based on this observation, as well as the colocalization of Vph1p and FM 4-64 with the internal structures, we concluded that IPEM2-GR cells contained numerous intact small vacuoles (Video 1) that can nevertheless coalesce to form a single larger vacuole when *FAB1* is deleted. In budding yeast cells, more than 130 genes, when defective, cause a fragmented/small vacuole phenotype (Michaillat & Mayer, 2013; Hurst & Fratti, 2020), but given the scope of this study, the cause of numerous small vacuoles in IPEM2-GR cells was not explored further.

To assess whether the small, clustered vacuole phenotype of IPEM2-GR cells was the result of enhanced endocytosis, we used FM 4-64 to label the plasma membrane of a mixture of IPEM2-GR and mNeon-Vph1-labeled WT cells, and simultaneously followed the endocytosis of FM 4–64 into cells over the course of 160 min. In this experiment, WT cells expressing mNeon-Vph1 were distinguishable from their IPEM2-GR counterparts by the presence of green vacuoles (Fig 2F and Video 2). Several FM 4-64–positive puncta of equal intensity were visible in both yeast strains beginning 20 min post–dye incubation (Fig 2F). By 40 min post-incubation, numerous FM 4-64–positive puncta were apparent in IPEM2-GR cells, and fewer, less intensely fluorescent puncta were visible in mNeon-Vph1–expressing cells (Fig 2F and Video 2). At 80 min post-incubation and time points beyond this, IPEM2-GR cells contained numerous clusters of small vacuoles that were far brighter in intensity than vacuoles in WT cells expressing mNeon-Vph1 (Fig 2F and Video 2). At the 10- and 20-min time points, FM 4-64–positive structures of similar intensity appear in both IPEM2-GR and WT cells expressing mNeon-Vph1 (Fig 2F), an observation that is consistent with similar rates of FM 4-64 uptake in these two strains. The similarity in the kinetics of FM 4-4 dye uptake is more readily apparent in early time points (∼2 min) (Video 2). Based on these data, we concluded that the rate of FM 4-64 uptake into IPEM2-GR cells was similar to that seen in mNeon-Vph1–expressing (and otherwise WT) cells. It is not immediately obvious what accounts for the increased FM 4-64 fluorescence intensity observed in IPEM2-GR cells.

Video 2 Rates of endocytosis in IPEM2-GR and WT cells are indistinguishable. IPEM2-GR and WT cells expressing Vph1-mNeon were labeled with FM 4-64 dye at 4°C, and after the removal of excess dye, cells were incubated in synthetic defined media at 25°C, and images were collected over the time duration indicated using a LSM 980 confocal microscope. Green channel: Vph1-mNeon; Red channel: FM 4-64. Download video

### IPEM2-GR cells use CME to reroute some plasma membrane proteins to vacuoles

If the stress response in IPEM2-GR cells was due to the arrival of unremodeled GPI-APs on the plasma membrane, a plausible outcome of this would be for cells to remove these proteins by endocytosis, as has been described for a misfolded GPI-AP in mammalian cells (Satpute-Krishan et al, 2014). However, the data presented in Fig 2C revealed that SNAPf-Gas1p was not endocytosed in IPEM2-GR cells. Therefore, we next asked whether IPEM2-GR cells altered the endocytosis of any other plasma membrane proteins.

**Figure 3.**
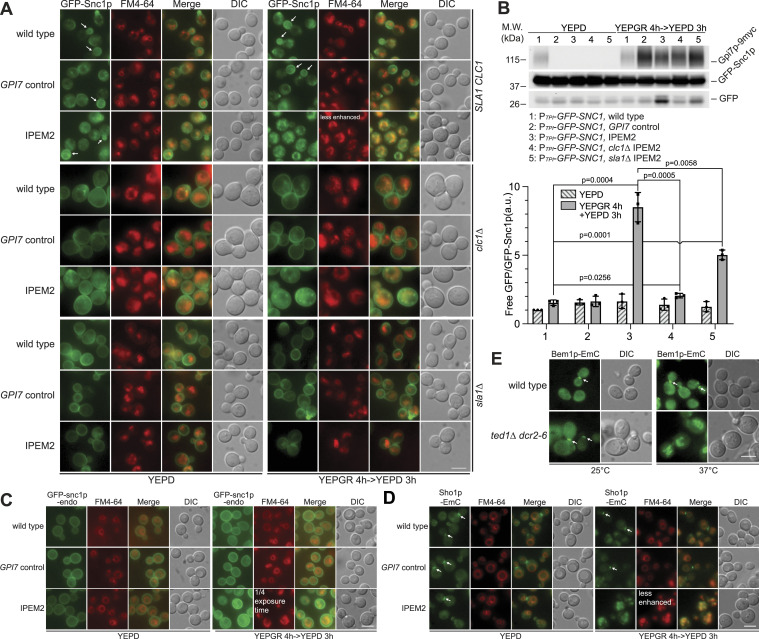
IPEM2-GR cells accumulate endocytosed proteins in numerous small vacuoles. **(A)** Clathrin-mediated trafficking repertoire of GFP-Snc1p is disrupted in IPEM2-GR cells. Note that *clc1Δ* cells are larger than their WT counterparts. **(B)** GFP-Snc1p is rerouted to the vacuole and degraded in IPEM2-GR cells. Upper panel: representative immunoblot of GFP-Snc1p from WCEs. n = 3; lower panel: quantification of the ratio between free GFP versus GFP-Snc1p from the various yeast strains (average + SD). n = 3. a.u. denotes arbitrary units. **(C)** GFP-snc1-endo is a poor substrate for endocytosis in IPEM2-GR cells. **(D)** In IPEM2-GR cells, Sho1p-EmC is redirected to numerous small vacuoles. EmC denotes enhanced monomeric Citrine. **(E)** In *ted1Δ dcr2-6* cells grown at 37°C, Bem1p-EmC was redirected to numerous small vacuoles. In all fluorescence panels, the scale bar is 5 μm.

To examine endocytosis in IPEM2-GR cells, we assessed the trafficking of the well-studied R-SNARE Snc1p (Lewis et al, 2000). In WT cells, Snc1p cycles between the Golgi, the plasma membrane, and the endosome, but at the steady state, the protein is predominantly localized to the plasma membrane, to nascent buds, and occasionally to internal puncta (Fig 3A) (Lewis et al, 2000). In IPEM-Glu cells and GPI7 cells grown in glucose (YEPD), the distribution of GFP-Snc1p was similar to that seen in WT cells. However, in IPEM2-GR cells, Snc1p was largely absent from the plasma membrane, accumulating instead in vacuoles, as judged by colocalization with FM 4-64 and the presence of “free” GFP in immunoblots (Fig 3A and B). GFP is resistant to vacuole-mediated proteolysis, and therefore, the presence of “free” GFP indicates that GFP-Snc1p reached the lumen of the vacuole. The absence of de novo–synthesized Snc1p from the plasma membrane of the daughter cell suggested that the trafficking of this protein between the endosome and the plasma membrane was defective in IPEM2-GR cells (Fig 3A).

The abnormal trafficking of GFP-Snc1p in IPEM2-GR cells was clathrin-mediated, as in IPEM2-GR cells lacking the light chain of clathrin (IPEM2 *clc1Δ*) or Sla1p (an effector of CME) (IPEM2 *sla1Δ*), GFP-Snc1p could be seen on the plasma membrane, as well as internal membranes (IPEM2-GR *clc1Δ*, Fig 3A). Given the apparent reduction of free GFP in immunoblots and the absence of any significant colocalization of these puncta with FM 4-64–positive membranes, we concluded that GFP-Snc1p was not efficiently delivered to the vacuole in IPEM2-GR *clc1Δ* cells. GFP-Snc1p was still able to reach the vacuole in IPEM2-GR *sla1Δ* cells, although at reduced levels compared with IPEM2-GR cells (Fig 3A and B). Interestingly, numerous GFP-Snc1p–positive internal structures could be seen in IPEM2-GR *clc1Δ* cells (Fig 3A). This population of GFP-Snc1p may have arisen via a clathrin-independent pathway and/or represent a pool of GFP-Snc1p in endosomes or the Golgi—but regardless of their origin, these membranous structures are poor substrates for fusion with the vacuole (Fig 3B).

Further evidence for the aberrant CME of GFP-Snc1p in IPEM2-GR cells was obtained by examining the localization of an endocytic mutant of Snc1p bearing substitutions of Val41 and Met43 to Ala (termed snc1p-endo; Lewis et al, 2000), which renders Snc1p unable to bind a clathrin adaptor. When expressed in IPEM2-GR cells, GFP-snc1p-endo was predominantly localized to the plasma membrane although some vacuolar fluorescence was also apparent (Fig 3C). The observation that trace amounts of GFP-snc1p-endo could still reach the vacuole in IPEM2-GR cells suggests either that newly synthesized protein was mislocalized to the vacuole from the Golgi or that a parallel endocytic pathway delivered GFP-snc1p-endo to the vacuole from the plasma membrane in IPEM2-GR cells.

In sum, we concluded that in IPEM2-GR cells, GFP-Snc1p could reach the plasma membrane, and thereafter entered the cell interior via CME.

The observation that the polarized distribution of GFP-Snc1p on the plasma membrane was defective in IPEM2-GR cells prompted us to seek evidence of additional proteins whose distributions might also be affected. For these experiments, we chose two SH3 domain–containing proteins with known roles in establishing yeast cell polarity and in responding to stress induced by osmotic changes—termed Bem1p and Sho1p, respectively (Leeuw et al, 1995; Tatebayashi et al, 2015). SH3 domains are commonly found in proteins subject to CME (Goode et al, 2015; Hummel & Kaksonen, 2023). Sho1p is a multispanning membrane protein, and in WT cells, Sho1p is found on the plasma membrane and at the bud neck (Fig 3D). In control cells and IPEM2-Glu cells, Sho1p localized to the bud neck, whereas in IPEM2-GR cells, Sho1p translocated to the vacuole (Fig 3D). Bem1p is a peripheral membrane protein that binds to PIP3 (Slessareva et al, 2006). Although Bem1p was localized to the bud neck in *ted1Δ dcr2–6* cells cultured at a temperature permissive for growth (25°C), the protein was found in the vacuole when *ted1Δ dcr2-6* cells were cultured at the growth-restrictive temperature (37°C) (Fig 3E).

**Figure S3.**
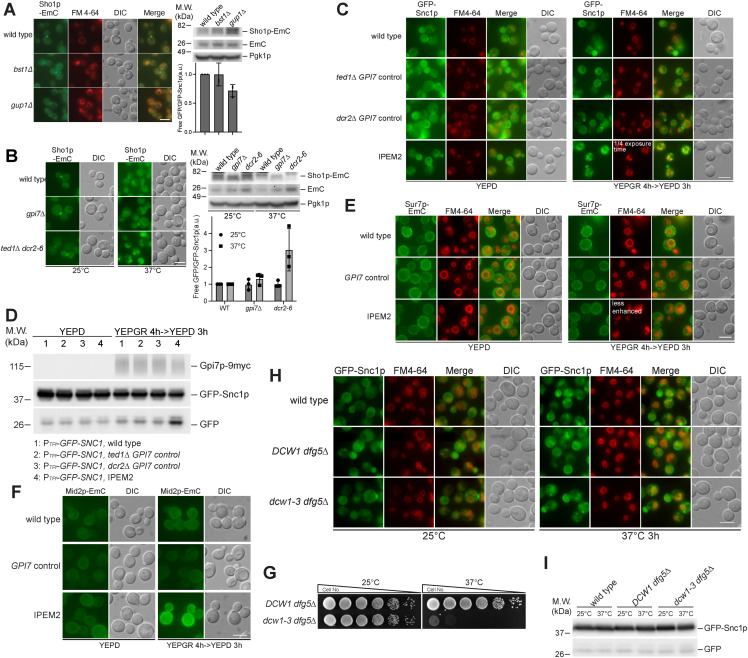
Endocytic trafficking of Sho1p, Snc1p, Sur7p, and Mid2p in GPI-AP remodeling mutants and in IPEM2-GR cells. **(A)** Left-hand side: Sho1p-EmC is not mislocalized to the vacuole in cells lacking the GPI-AP remodelase genes *BST1* and *GUP1*. The scale bar is 5 μm. Right-hand side: quantification of the ratio between Sho1p-EmC versus free EmC, n = 3. Pgk1p serves as a gel loading control. **(B)** Left-hand side: Sho1p-EmC is largely restricted to the bud neck and limiting membrane of the vacuole in cells lacking *GPI7* grown at 37°C but localizes to numerous small vacuoles in IPEM2-GR cells. The scale bar is 5 μm. Right-hand side: quantification of the ratio between Sho1p-EmC versus free EmC, n = 3. Pgk1p serves as a gel loading control. **(C)** Trafficking repertoire of GFP-Snc1p in cells lacking either *TED1* or *DCR2* is indistinguishable from that in WT cells. The scale bar is 5 μm. **(D)** GFP-Snc1p is largely excluded from the lumen of the vacuole in cells lacking either *TED1* or *DCR2*. **(E)** Intracellular distribution of the eisosome component Sur7p-EmC is unaffected in IPEM2-GR cells. The scale bar is 5 μm. **(F)** Intracellular distribution of the cell wall integrity pathway sensor Mid2p-EmC is unaffected in IPEM2-GR cells. Note that, as reported previously (Chen et al, 2021), the expression level of Mid2p-EmC is increased in IPEM2-GR cells. The scale bar is 5 μm. **(G)** *dcw1-3 dfg5Δ* cells are temperature-sensitive for growth. The 10-fold serial dilutions of the indicated strains were spotted onto YEPD plates, which were thereafter incubated at 25 or 37°C for 3 d before being photographed. **(H)** Trafficking repertoire of GFP-Snc1p is unaffected in *dcw1-3 dfg5Δ* cells grown at 37°C. The scale bar is 5 μm. **(I)** GFP-Snc1p is not degraded in *dcw1-3 dfg5Δ* cells grown at 37°C.

Importantly, the redirection in Snc1p, Bem1p, and Sho1p from the plasma membrane to the vacuole in Man2 remodeling mutants is specific to the deficiency in the removal of EtNP on Man2 of GPI-APs. This was evident from the fact that deletion of the GPI-AP lipid remodeling genes *BST1* (Fujita et al, 2006), *GUP1* (Bosson et al, 2006), or *GPI7* (which adds EtNP on Man2) did not result in the mislocalization of Sho1p to vacuoles (Fig S3A and B). Similarly, deletion of *TED1* (which removes EtNP from Man2 in the ER) or *DCR2* also did not lead to mislocalization of GFP-Snc1p to the vacuole (Fig S3C and D). These observations are significant as cells lacking *TED1*, *GUP1*, and *BST1* exhibit a delay in the ER export of Gas1p (Chen & Banfield, 2022; Rodriguez-Gallardo et al, 2022), revealing that the phenotypes we observe in this study for IPEM2-GR cells are not a consequence of an accumulation of Gas1p (or indeed other GPI-APs) in the ER. Not all the plasma membrane proteins we examined were endocytosed in IPEM2-GR cells, the distributions of Sur7p (a component of the eisosome; Walther et al, 2006) and Mid2p (a sensor of the cell wall integrity pathway; Rajavel et al, 1999) were unaltered (Fig S3E and F). Eisosomes mark stable sites of endocytosis, whereas Sur7p is important for CME. Sur7p is a long-lived protein that remains stably associated with the eisosome (Walther et al, 2006; Thayer et al, 2014). In contrast, Mid2p binds to AP-2 and as such is a substrate for CME albeit under certain stress conditions (Chapa-y-Lazo et al, 2014). Despite IPEM2-GR cells being stress-activated, Mid2p was not endocytosed (Fig S3F).

In addition, in temperature-sensitive dcw*1–3 dfg5Δ* cells (Fig S3G), which fail to transfer the GPI-APs from the plasma membrane to the cell wall when grown at 37°C (Kitagaki et al, 2002, 2004; Vogt et al, 2020), the polarized distribution and degradation of GFP-Snc1p were not affected (Fig S3H and I).

In sum, these data suggest that it is the presence of EtNP on Man2 of GPI-APs, rather than the failure to cleave GPI-APs at the plasma membrane, that accounts for the redirection of Snc1p, Sho1p, and Bem1p to the vacuole in IPEM-GR cells.

### Ubiquitination is a prerequisite for abnormal endocytosis in IPEM2-GR cells

Plasma membrane proteins destined for degradation are transported into the interior of the vacuole via the multivesicular body (MVB) (Babst, 2011). The formation of the MVB involves the function of a series of ESCRT complexes that act successively (Babst, 2011). To explore a role of the MVB in the degradation of GFP-Snc1p, we deleted *VPS27* or *DOA4* in the IPEM2 strain. Vps27p is a component of the ESCRT-0 complex and mediates protein degradation by binding to and incorporating proteins that have been ubiquitinated into the nascent MVB (Katzmann et al, 2003), whereas Doa4p is a ubiquitin hydrolase that functions to recycle ubiquitin at a later stage in MVB biogenesis (Nikko & André, 2007).

**Figure 4.**
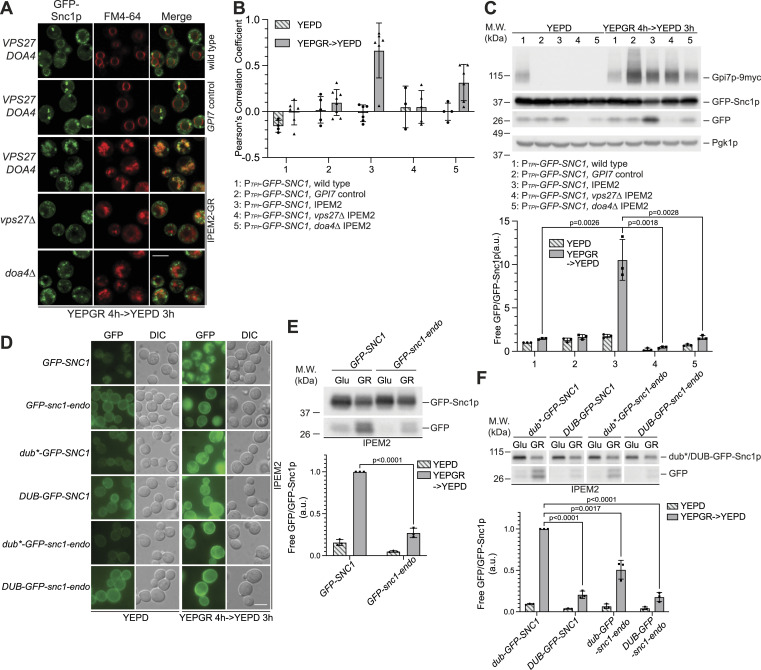
Ubiquitination is a prerequisite for the abnormal endocytosis observed in IPEM2-GR cells. **(A)** GFP-Snc1p is rerouted numerous small vacuoles in IPEM2-GR cells via the MVB pathway. The scale bar is 5 μm. Images were acquired using a LSM 980 confocal microscope. **(A, B)** Colocalization of GFP-Snc1p puncta and FM 4-64–labeled membranes from the experiment depicted in (A). The arrowheads and dots indicate the number of cells scored. **(C)** A functional ESCRT pathway is required for delivery of GFP-Snc1p to the vacuole in IPEM2-GR cells. The scale bar is 5 μm. Upper panel: representative immunoblot from WCEs. n = 3; lower panel: quantification of the ratio between free GFP versus GFP-Snc1p (average + SD). n = 3. a.u. denotes arbitrary units. Pgk1p serves as a gel loading control. **(D)** Endocytosis of GFP-Snc1p in IPEM2-GR cells is ubiquitin-dependent. DUB denotes the de-ubiquitination domain from Ubp7p and dub* the de-ubiquitination–deficient domain mutant. **(E)** Endocytosis-deficient mutant of GFP-Snc1p (GFP-snc1-endo) is a poor substrate for delivery to the vacuole in IPEM2-GR cells. a.u. denotes arbitrary units. Upper panel: representative immunoblot of GFP-Snc1p from WCEs. n = 3; lower panel: quantification of the ratio between free GFP versus GFP-Snc1p (average + SD). n = 3. a.u. denotes arbitrary units. **(F)** Ubiquitination of GFP-Snc1p is a prerequisite for delivery to the vacuole in IPEM2-GR cells. Upper panel: representative immunoblot of GFP-Snc1p from WCEs. n = 3; lower panel: quantification of the ratio between free GFP versus GFP-Snc1p (average + SD). n = 3. a.u. denotes arbitrary units. In all fluorescence panels, the scale bar is 5 μm.

To assess the impact of deletion of *VPS27* or *DOA4* in IPEM2-GR cells, we examined the fate of GFP-Snc1p by fluorescence microscopy and monitored the degradation of GFP-Snc1p by immunoblotting of WCEs. In IPEM2-GR cells, GFP-Snc1p accumulated in internal puncta and in vacuoles (as judged by colocalization with an FM 4-64 dye) (Fig 4A). In contrast, in IPEM2-GR cells in which either *VPS27* or *DOA4* was deleted (IPEM2-GR *vps27Δ* and IPEM2-GR *doa4Δ*), most of GFP-Snc1p was found in internal structures that did not colocalize with an FM 4-64 dye (Fig 4A and B). Consistent with the fluorescence microscopy data, immunoblotting of WCEs revealed that when either *VPS27* or *DOA4* was deleted from IPEM2-GR cells (IPEM2 *vps27Δ* and IPEM2 *doa4Δ*), less protease-resistant GFP was evident, compared with IPEM2-GR cells (Fig 4C). These data suggest that disrupting the formation of MVBs in IPEM2-GR cells prevented delivery of GFP-Snc1p to the vacuole lumen.

**Figure S4.**
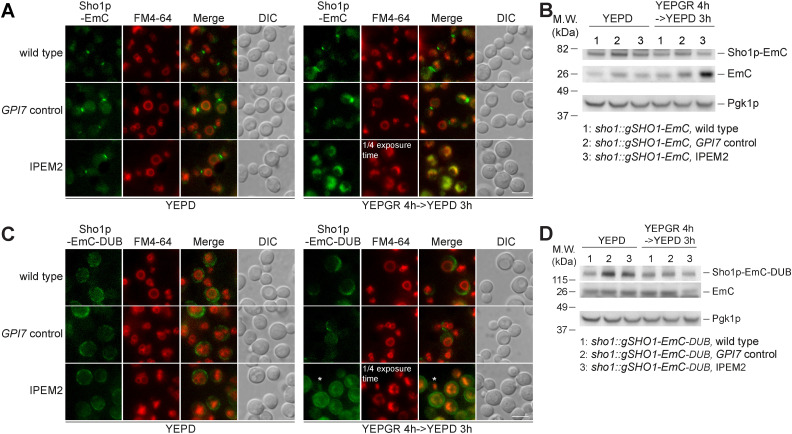
Ubiquitination of Sho1p-EmC is a prerequisite for the protein’s endocytosis in IPEM2-GR cells. **(A, B)** Sho1p-EmC is mislocalized to and degraded in numerous small vacuoles in IPEM2-GR cells. **(C, D)** De-ubiquitin domain Sho1p-EmC fusion protein Sho1p-EmC-DUB is largely localized to the plasma membrane in IPEM2-GR cells. In panels (B, D), the cytoplasmic protein Pgk1p serves as a gel loading control.

To more directly assess the role of ubiquitin in the endocytosis of Snc1p, we fused the de-ubiquitination domain of Ubp7p or the de-ubiquitination–deficient domain (ubp7p; C618S) to GFP-Snc1p, generating DUB-GFP-Snc1p or dub*-GFP-Snc1p. Similar replacements were also made to the endocytosis-deficient form of Snc1p (snc1-endo) generating DUB-GFP- snc1-endo and dub*-GFP-snc1-endo (Fig 4) (Stringer & Piper, 2011). The GFP-Snc1p fusion proteins were introduced into IPEM2-GR cells, and the fate of fusion proteins was examined by fluorescence microscopy and immunoblotting (Fig 4D–F, respectively). As expected, DUB-GFP-Snc1p was uniformly distributed on the plasma membrane, and comparatively little protease-resistant GFP was apparent in WCEs from IPEM2-GR cells—data that are consistent with ubiquitination being a prerequisite for the endocytosis of Snc1p (Fig 4D and F). In contrast, dub*-GFP-Snc1p localized to both the plasma membrane and internal structures, and comparatively more protease-resistant GFP (∼75% more) was apparent in WCEs from these cells compared with DUB-GFP-Snc1p (Fig 4D and F). Although GFP-snc1-endo was a poor substrate for CME (Figs 3C and 4D), ubiquitination was still required for its endocytosis (Fig 4D and F). Similar findings were also obtained with Sho1p-EmC-DUB in IPEM2-GR cells (Fig S4). In IPEM2-Glu cells, Sho1p-EmC was primarily found at the mother/daughter cell junction (Fig S4A and B), whereas Sho1p-EmC localized to the highly fragmented vacuoles in IPEM2-GR cells (Fig S4A and B). Sho1p-EmC-DUB was no longer found at the mother/daughter cell junction in control cells (grown in glucose or galactose + raffinose) or in IPEM2-Glu cells (Fig S4C and D). Instead, Sho1p-EmC-DUB was distributed non-uniformly on the plasma membrane, revealing a role of ubiquitination in the redirection of Sho1p to the mother/daughter cell junction (Fig S4C). In IPEM2-GR cells, Sho1p-EmC-DUB was found on the plasma membrane (although the protein appeared to be largely excluded from the daughter cell plasma membrane) and in the numerous small vacuoles of both the mother and daughter cells (Fig S4C).

### The E3 ligase Rsp5p plays multiple roles in the endocytosis of proteins in IPEM2-GR cells

**Figure 5.**
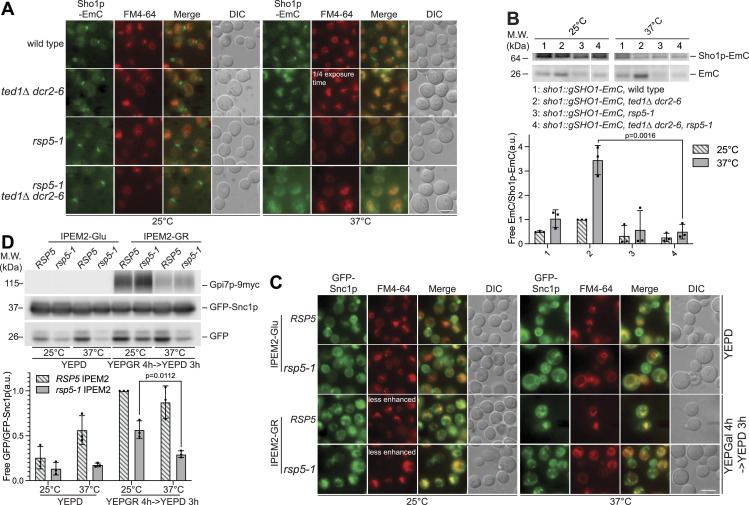
Rsp5p plays multiple roles in the endocytosis of proteins in GPI-AP Man2 remodeling mutants. **(A)** Sho1p-EmC reaches the limiting membrane of the vacuole in *rsp5-1 ted1Δ dcr2-6* cells grown at 37°C. The scale bar is 5 μm. **(B)** Sho1p-EmC is not internalized and degraded in the vacuole in *rsp5-1 ted1Δ dcr2-6* cells grown at 37°C. Immunoblot of WCEs from the indicated strains grown at 25 or 37°C. Pgk1p serves as a gel loading control, n = 3. **(C)** Endocytosis and internalization of GFP-Snc1p into the vacuole are diminished in *rsp5-1* IPEM2-GR cells grown at 37°C. Note that the fragmented vacuole phenotype evident in IPEM2-GR cells is largely absent in *rsp5-1* IPEM2-GR cells grown at 37°C. The scale bar is 5 μm. **(D)** Immunoblot of WCEs from the indicated strains grown at 25 or 37°C. Pgk1p serves as a gel loading control, n = 3. Note that the degradation of GFP-Snc1p is significantly reduced in *rsp5-1* IPEM2-GR cells grown at 37°C.

*RSP5* encodes a NEDD4 family E3 ubiquitin ligase implicated in multiple processes including the regulation of endocytosis and the sorting of proteins into the MVB (Katzmann et al, 2004). To examine the role of Rsp5p in the ubiquitin-dependent endocytosis of Sho1p, we introduced a temperature-sensitive allele of *RSP5* (*rsp5-1*) (Dunn & Hicke, 2001; Katzmann et al, 2003) into *ted1Δ dcr2-6* cells. Although *rsp5-1* did not affect the localization of Sho1p-EmC grown at 37°C in otherwise WT cells, in *rsp5-1 ted1Δ dcr2–6* cells grown at 37°C, Sho1p-EmC localized to the limiting membrane of the vacuole (Fig 5A) and was excluded from the vacuole lumen, as evidenced by the absence of free GFP in immunoblots of WCEs from these cells (Fig 5B). These data indicate that Rsp5p activity is not required for the initial stages of endocytosis of Sho1p in the temperature-sensitive Man2 remodeling mutant, but rather is necessary for the internalization and subsequent degradation of Sho1p in the vacuole. In contrast to Sho1p, Snc1p required Rsp5p activity both for the initial stages of endocytosis and for the internalization and degradation of Snc1p in the vacuole (Ma & Burd, 2019). However, we cannot exclude the possibility that the differences observed for cargoes and Rsp5p activity are not affected by the genetic background of the strains used. In IPEM2-GR *rsp5-1* cells, Snc1p was still observable on the plasma membrane, and comparatively less GFP-Snc1p was delivered to the vacuole as indicated by the reduction in the amount of “free” GFP in immunoblots (Fig 5C and D). Interestingly, the introduction of the *rsp5-1* allele into *ted1Δ dcr2-6* cells substantially reduced the numerous small vacuole phenotype of these cells at 37°C (FM 4-64 staining; Fig 5A and C), suggesting that this phenotype arises from an imbalance between the fusion and fission of vacuolar membranes.

## Discussion

The plasma membrane is comprised of non-symmetrical distributions of various lipids and membrane proteins. However, the extent to which the formation of lipid domains on the plasma membrane is believed to involve the sequestration and subsequent enrichment of certain lipids (e.g., sphingolipids and sterols) has yet to be resolved. This uncertainty also applies to the role of and/or partitioning of GPI-APs within the plasma membrane (Simons & Toomre, 2000; Lingwood & Simons, 2007; Simons & Sampaio, 2011; Sevcsik et al, 2015). Nevertheless, the segregation of membrane proteins in the plasma membrane is thought to play an important role in the creation of platforms that can mediate a variety of signaling events—activities that are critical to the cell’s capacity to respond to a variety of stimuli and as such vital for cellular adaptation and survival (Szpurka et al, 2008; Lakhan et al, 2009; Levental et al, 2010; Suzuki et al, 2012; Sharonov et al, 2016; Zeng et al, 2023).

Although it is well documented that the removal of EtNP from Man2 functions as a transport warrant for the robust export of GPI-APs from the ER (Manzano-Lopez et al, 2015; Rodriguez-Gallardo et al, 2022), there is currently limited information regarding any further consequences of evading this remodeling event (Chen et al, 2021). In this study, we set out to further delineate the cell biological consequences of Man2 unremodeled GPI-APs trafficked to the plasma membrane of budding yeast cells. We show that in addition to the activation of the SAC and the cell wall integrity pathway (Chen et al, 2021), Man2 unremodeled GPI-APs also trigger abnormal endocytosis of certain membrane proteins and lead to the formation of numerous small vacuoles. Given the evolutionary ubiquity of the Man2 remodeling event, our findings may be broadly applicable to GPI-APs in other eukaryotes.

Presumably, the presence of atypically larger numbers of unremodeled GPI-APs in the plasma membrane triggers a stress response. Man2 unremodeled GPI-APs in the plasma membrane may disrupt signaling platforms or lead to a perceived excess of GPI-APs outside of signaling platforms, which may impact membrane homeostasis (Fig 1H and I). In this regard, we note that the segregation of another so-called lipid raft protein (Pma1p) into DRMs was not affected in IPEM2-GR cells (Fig 1D) and that the signaling response pathway leading to activation of the cell wall integrity pathway also remained intact (Posas et al, 1998; Chen et al, 2021).

Despite the observation that Gas1p is not endocytosed (Fig 2C) in IPEM2-GR cells, we cannot exclude the possibility that other yeast GPI-APs may be endocytosed (Yin et al, 2005). Indeed, several yeast GPI-APs are subject to robust endocytosis and trafficking to the vacuole (MacDonald et al, 2015). As such, it seems most plausible that the phenotypes we have characterized here and elsewhere (Chen et al, 2021) for the IPEM2-GR mutant most likely arise from the trafficking and delivery of GPI-APs bearing EtNP on Man2 to the plasma membrane. Consistent with this, we found that IPEM2-GR cells exhibit an increase in membrane disorder when grown under conditions where EtNP is not removed from Man2 of GPI-APs (Fig 1H and I).

Yeast mutants defective in a mannosidase that transfers the GPI-linked protein to the cell wall reportedly exhibit a cell cycle phenotype, wherein cells arrest growth with small buds (Kitagaki et al, 2004). Similarly, *cdc1* mutants, which accumulate GPI-APs bearing EtNP on Man1, also arrest growth with small buds (Hartwell, 1971; Vazquez et al, 2014; Yang & Banfield, 2020). However, cells carrying mutations in mannosidases that cleave GPI-APs do not phenocopy IPEM2-GR cells (Fig S3G–I). These data are in accord with our presumption that it is the failure to remodel GPI-APs, rather than defects in GPI-AP cleavage, that generates the plasma membrane stress signal. Indeed, *cdc1* mutants display many of the phenotypes we have characterized for IPEM2-GR cells, which can perhaps best be reconciled with the view that Man1 unremodeled GPI-APs on the plasma membrane also elicit a stress response (Chen, Li, Lau, Yang, and Banfield, unpublished observations).

Is the abnormal endocytosis observed in IPEM2-GR cells triggered in direct response to perturbations in the plasma membrane, or as part of an archetypal response whose purpose is to reset the organization and/or compositional status of the plasma membrane? Universal clearance of Man2 unremodeled GPI-APs from the plasma membrane may not be the objective of the stress-induced abnormal CME we uncover here, as at least one GPI-AP, Gas1p, was not endocytosed (Fig 2C). Besides, others have shown some GPI-APs undergo robust endocytosis when properly remodeled (MacDonald et al, 2015). Furthermore, the fate of proteins that are endocytosed in IPEM2-GR cells (Bem1p, Sho1p, and Snc1p) is distinct from that which occurs in otherwise WT cells (Figs 3 and 4 and S3). In WT cells, Bem1p and Sho1p are rerouted from the plasma membrane to the bud neck, whereas Snc1p cycles between the plasma membrane and the nascent bud. In IPEM2-GR cells, Bem1p, Sho1p, and Snc1p are endocytosed and redirected to the vacuole for degradation via the MVB (Figs 3 and 4 and S3). By virtue of their SH3 domains, Bem1p and Sho1p are likely substrates for CME. Interestingly, Mid2p is also a substrate for CME, and yet, Mid2p is not endocytosed in IPEM-GR cells (Fig S3). It seems plausible that the endocytic phenomena observed in IPEM2-GR cells may have at least two purposes: to re-establish plasma membrane homeostasis and to transmit stress signals to the interior of the cell (Chen et al, 2021). The stress signal may rely on, or be comprised of, a subset of proteins and/or lipids that are selected for endocytosis in IPEM2-GR cells.

## Materials and Methods

*Saccharomyces cerevisiae* strains used in this study are listed in Table 1, plasmids used in this study are listed in Table 2, and antibodies used in this study are listed in Table 3.

**Table 1.** Strains.

Strains	Description	Source
Fig 1B		
SARY5529	MATa; his3∆1; leu2∆0; met15∆0; ura3∆0; gGPI7-9MYC	DKB Lab Collection
SARY5768	MATa; his3∆1; leu2∆0; met15∆0; ura3∆0; PGPI7::PGAL gpi7::GPI7-9MYC	DKB Lab Collection
SARY5769	MATa; his3∆1; leu2∆0; met15∆0; ura3∆0; PGPI7::PGAL gpi7::GPI7-9MYC; ted1::kanMX4; dcr2(-255-1490)::LoxP	DKB Lab Collection
Fig 1C		
SARY7333	MATa; his3∆1; leu2∆0; met15∆0; ura3∆0; PGPI7::PGAL gpi7::GPI7-9MYC; PGAS1(-461- -11)::K.l.LEU2; pRS406-PGALs-mNeon-GAS1	DKB Lab Collection
Fig 1D		
SARY7335	MATa; his3∆1; leu2∆0; met15∆0; ura3∆0; PGPI7::PGAL gpi7::GPI7-9MYC; ted1::kanMX4; dcr2(-255-1490)::LoxP; PGAS1(-461- -11)::K.l.LEU2; pRS406-PGALs-mNeon-GAS1	DKB Lab Collection
Fig 1H		
SARY5529	MATa; his3∆1; leu2∆0; met15∆0; ura3∆0; gGPI7-9MYC	DKB Lab Collection
SARY5768	MATa; his3∆1; leu2∆0; met15∆0; ura3∆0; PGPI7::PGAL gpi7::GPI7-9MYC	DKB Lab Collection
SARY8531	MATa; his3∆1; leu2∆0; met15∆0; ura3∆0; PGPI7::PGAL gpi7::GPI7-9MYC; ted1::HIS3MX6	DKB Lab Collection
SARY5769	MATa; his3∆1; leu2∆0; met15∆0; ura3∆0; PGPI7::PGAL gpi7::GPI7-9MYC; ted1::kanMX4; dcr2(-255-1490)::LoxP	DKB Lab Collection
Fig 2A		
SARY6839	MATa; his3∆1; leu2∆0; met15∆0; ura3∆0; gGPI7-9MYC; mScarlet-gGAS1; pRS406-PGALs-mNeon-GAS1	DKB Lab Collection
SARY6841	MATa; his3∆1; leu2∆0; met15∆0; ura3∆0; PGPI7::PGAL gpi7::GPI7-9MYC; mScarlet-gGAS1; pRS406-PGALs-mNeon-GAS1	DKB Lab Collection
SARY6845	MATa; his3∆1; leu2∆0; met15∆0; ura3∆0; PGPI7::PGAL gpi7::GPI7-9MYC; ted1::kanMX4; dcr2(-255-1490)::LoxP; mScarlet-gGAS1; pRS406-PGALs-mNeon-GAS1	DKB Lab Collection
Fig 2B		
SARY8005	MATa; his3∆1; leu2∆0; met15∆0; ura3∆0; gGPI7-9MYC; pRS406-PGALs-SNAPf-GAS1	DKB Lab Collection
SARY8007	MATa; his3∆1; leu2∆0; met15∆0; ura3∆0; PGPI7::PGAL gpi7::GPI7-9MYC; pRS406-PGALs- SNAPf -GAS1	DKB Lab Collection
SARY8009	MATa; his3∆1; leu2∆0; met15∆0; ura3∆0; PGPI7::PGAL gpi7::GPI7-9MYC; ted1::kanMX4; dcr2(-255-1490)::LoxP; pRS406-PGALs- SNAPf -GAS1	DKB Lab Collection
Fig 2C		
SARY8191	MATa; his3∆1; leu2∆0; met15∆0; ura3∆0; PGPI7::PGAL gpi7::GPI7-9MYC; ted1::kanMX4; dcr2(-255-1490)::LoxP; vph1::VPH1-mNeon-K.l. LEU2	DKB Lab Collection
Fig 2D and E		
SARY8194	MATa; his3∆1; leu2∆0; met15∆0; ura3∆0; gGPI7-9MYC, fab1::HIS3MX6	DKB Lab Collection
SARY8195	MATa; his3∆1; leu2∆0; met15∆0; ura3∆0; PGPI7::PGAL gpi7::GPI7-9MYC, fab1::HIS3MX6	DKB Lab Collection
SARY8198	MATa; his3∆1; leu2∆0; met15∆0; ura3∆0; PGPI7::PGAL gpi7::GPI7-9MYC; ted1::HIS3MX6, fab1::HIS3MX6	DKB Lab Collection
Fig 2F		
SARY8187	MATa; his3∆1; leu2∆0; met15∆0; ura3∆0; gGPI7-9MYC; vph1::VPH1-mNeon-K.l. LEU2	DKB Lab Collection
SARY5769	MATa; his3∆1; leu2∆0; met15∆0; ura3∆0; PGPI7::PGAL gpi7::GPI7-9MYC; ted1::kanMX4; dcr2(-255-1490)::LoxP	DKB Lab Collection
Fig 3A		
SARY7782	MATa; his3∆1; leu2∆0; met15∆0; ura3∆0; gGPI7-9MYC; pRS406-PTPI1- GFP-SNC1-CYC1ter	DKB Lab Collection
SARY7784	MATa; his3∆1; leu2∆0; met15∆0; ura3∆0; PGPI7::PGAL gpi7::GPI7-9MYC; pRS406-PTPI1- GFP-SNC1-CYC1ter	DKB Lab Collection
SARY7786	MATa; his3∆1; leu2∆0; met15∆0; ura3∆0; PGPI7::PGAL gpi7::GPI7-9MYC; ted1::kanMX4; dcr2(-255-1490)::LoxP; pRS406-PTPI1- GFP-SNC1-CYC1ter	DKB Lab Collection
SARY7858	MATa; his3∆1; leu2∆0; met15∆0; ura3∆0; gGPI7-9MYC; pRS406-PTPI1- GFP-SNC1-CYC1ter; clc1::HIS3MX6	DKB Lab Collection
SARY7876	MATa; his3∆1; leu2∆0; met15∆0; ura3∆0; PGPI7::PGAL gpi7::GPI7-9MYC; pRS406-PTPI1- GFP-SNC1-CYC1ter; clc1::HIS3MX6	DKB Lab Collection
SARY7879	MATa; his3∆1; leu2∆0; met15∆0; ura3∆0; PGPI7::PGAL gpi7::GPI7-9MYC; ted1::kanMX4; dcr2(-255-1490)::LoxP; pRS406-PTPI1- GFP-SNC1-CYC1ter; clc1::HIS3MX6	DKB Lab Collection
SARY7916	MATa; his3∆1; leu2∆0; met15∆0; ura3∆0; gGPI7-9MYC; pRS406-PTPI1- GFP-SNC1-CYC1ter; sla1::HIS3MX6	DKB Lab Collection
SARY7818	MATa; his3∆1; leu2∆0; met15∆0; ura3∆0; PGPI7::PGAL gpi7::GPI7-9MYC; pRS406-PTPI1- GFP-SNC1-CYC1ter; sla1::HIS3MX6	DKB Lab Collection
SARY7920	MATa; his3∆1; leu2∆0; met15∆0; ura3∆0; PGPI7::PGAL gpi7::GPI7-9MYC; ted1::kanMX4; dcr2(-255-1490)::LoxP; pRS406-PTPI1- GFP-SNC1-CYC1ter; sla1::HIS3MX6	DKB Lab Collection
Fig 3B		
SARY7782	MATa; his3∆1; leu2∆0; met15∆0; ura3∆0; gGPI7-9MYC; pRS406-PTPI1- GFP-SNC1-CYC1ter	DKB Lab Collection
SARY7784	MATa; his3∆1; leu2∆0; met15∆0; ura3∆0; PGPI7::PGAL gpi7::GPI7-9MYC; pRS406-PTPI1- GFP-SNC1-CYC1ter	DKB Lab Collection
SARY7786	MATa; his3∆1; leu2∆0; met15∆0; ura3∆0; PGPI7::PGAL gpi7::GPI7-9MYC; ted1::kanMX4; dcr2(-255-1490)::LoxP; pRS406-PTPI1- GFP-SNC1-CYC1ter	DKB Lab Collection
SARY7879	MATa; his3∆1; leu2∆0; met15∆0; ura3∆0; PGPI7::PGAL gpi7::GPI7-9MYC; ted1::kanMX4; dcr2(-255-1490)::LoxP; pRS406-PTPI1- GFP-SNC1-CYC1ter; clc1::HIS3MX6	DKB Lab Collection
SARY7920	MATa; his3∆1; leu2∆0; met15∆0; ura3∆0; PGPI7::PGAL gpi7::GPI7-9MYC; ted1::kanMX4; dcr2(-255-1490)::LoxP; pRS406-PTPI1- GFP-SNC1-CYC1ter; sla1::HIS3MX6	DKB Lab Collection
Fig 3C		
SARY7788	MATa; his3∆1; leu2∆0; met15∆0; ura3∆0; gGPI7-9MYC; pRS406-PTPI1- GFP-snc1-endo-CYC1ter	DKB Lab Collection
SARY7790	MATa; his3∆1; leu2∆0; met15∆0; ura3∆0; PGPI7::PGAL gpi7::GPI7-9MYC; pRS406-PTPI1- GFP-snc1-endo-CYC1ter	DKB Lab Collection
SARY7792	MATa; his3∆1; leu2∆0; met15∆0; ura3∆0; PGPI7::PGAL gpi7::GPI7-9MYC; ted1::kanMX4; dcr2(-255-1490)::LoxP; pRS406-PTPI1- GFP-snc1-endo-CYC1ter	DKB Lab Collection
Fig 3D		
SARY7197	MATa; his3∆1; leu2∆0; met15∆0; ura3∆0; gGPI7-9MYC; sho1::SHO1-EmCitrine-K.l. LEU2	DKB Lab Collection
SARY7199	MATa; his3∆1; leu2∆0; met15∆0; ura3∆0; PGPI7::PGAL gpi7::GPI7-9MYC; sho1::SHO1-EmCitrine-K.l. LEU2	DKB Lab Collection
SARY7201	MATa; his3∆1; leu2∆0; met15∆0; ura3∆0; PGPI7::PGAL gpi7::GPI7-9MYC; ted1::kanMX4; dcr2(-255-1490)::LoxP; sho1::SHO1-EmCitrine-K.l. LEU2	DKB Lab Collection
Fig 3E		
SARY5087	MATa; his3∆1; leu2∆0; met15∆0; ura3∆0; bem1::BEM1-EmCitrine-K.l. LEU2	DKB Lab Collection
SARY5091	MATa; his3∆1; leu2∆0; met15∆0; ura3∆0; ted1::kanMX4; dcr2(-255-1490)::LoxP; pRS413-gdcr2-6; bem1::BEM1-EmCitrine-K.l. LEU2	DKB Lab Collection
Fig 4A–C		
SARY7782	MATa; his3∆1; leu2∆0; met15∆0; ura3∆0; gGPI7-9MYC; pRS406-PTPI1- GFP-SNC1-CYC1ter	DKB Lab Collection
SARY7784	MATa; his3∆1; leu2∆0; met15∆0; ura3∆0; PGPI7::PGAL gpi7::GPI7-9MYC; pRS406-PTPI1- GFP-SNC1-CYC1ter	DKB Lab Collection
SARY7786	MATa; his3∆1; leu2∆0; met15∆0; ura3∆0; PGPI7::PGAL gpi7::GPI7-9MYC; ted1::kanMX4; dcr2(-255-1490)::LoxP; pRS406-PTPI1- GFP-SNC1-CYC1ter	DKB Lab Collection
SARY8367	MATa; his3∆1; leu2∆0; met15∆0; ura3∆0; PGPI7::PGAL gpi7::GPI7-9MYC; ted1::kanMX4; dcr2(-255-1490)::LoxP; pRS406-PTPI1- GFP-SNC1-CYC1ter; doa4::HIS3MX6	DKB Lab Collection
SARY8369	MATa; his3∆1; leu2∆0; met15∆0; ura3∆0; PGPI7::PGAL gpi7::GPI7-9MYC; ted1::kanMX4; dcr2(-255-1490)::LoxP; pRS406-PTPI1- GFP-SNC1-CYC1ter; vps27::HIS3MX6	DKB Lab Collection
Fig 4D–F		
SARY7786	MATa; his3∆1; leu2∆0; met15∆0; ura3∆0; PGPI7::PGAL gpi7::GPI7-9MYC; ted1::kanMX4; dcr2(-255-1490)::LoxP; pRS406-PTPI1- GFP-SNC1-CYC1ter	DKB Lab Collection
SARY7792	MATa; his3∆1; leu2∆0; met15∆0; ura3∆0; PGPI7::PGAL gpi7::GPI7-9MYC; ted1::kanMX4; dcr2(-255-1490)::LoxP; pRS406-PTPI1- GFP-snc1-endo-CYC1ter	DKB Lab Collection
SARY8396	MATa; his3∆1; leu2∆0; met15∆0; ura3∆0; PGPI7::PGAL gpi7::GPI7-9MYC; ted1::kanMX4; dcr2(-255-1490)::LoxP; pRS406-PTPI1- DUB-GFP-SNC1-CYC1ter	DKB Lab Collection
SARY8400	MATa; his3∆1; leu2∆0; met15∆0; ura3∆0; PGPI7::PGAL gpi7::GPI7-9MYC; ted1::kanMX4; dcr2(-255-1490)::LoxP; pRS406-PTPI1- dub*-GFP-SNC1-CYC1ter	DKB Lab Collection
SARY8613	MATa; his3∆1; leu2∆0; met15∆0; ura3∆0; PGPI7::PGAL gpi7::GPI7-9MYC; ted1::kanMX4; dcr2(-255-1490)::LoxP; pRS406-PTPI1- DUB-GFP-snc1-endo-CYC1ter	DKB Lab Collection
SARY8615	MATa; his3∆1; leu2∆0; met15∆0; ura3∆0; PGPI7::PGAL gpi7::GPI7-9MYC; ted1::kanMX4; dcr2(-255-1490)::LoxP; pRS406-PTPI1- dub*-GFP-snc1-endo-CYC1ter	DKB Lab Collection
Fig 5A and B		
SARY5468	MATa; his3∆1; leu2∆0; met15∆0; ura3∆0; sho1::SHO1-EmCitrine-K.l. LEU2	DKB Lab Collection
SARY5492	MATa; his3∆1; leu2∆0; met15∆0; ura3∆0; ted1::kanMX4; dcr2(-255-1490)::LoxP; pRS413-gdcr2-6; sho1::SHO1-EmCitrine-K.l. LEU2	DKB Lab Collection
SARY8324	MATa; his3∆1; leu2∆0; met15∆0; ura3∆0; sho1::SHO1-EmCitrine-K.l. LEU2; rsp5::rsp5-1-K.l. URA3	DKB Lab Collection
SARY8326	MATa; his3∆1; leu2∆0; met15∆0; ura3∆0; ted1::kanMX4; dcr2(-255-1490)::LoxP; pRS413-gdcr2-6; sho1::SHO1-EmCitrine-K.l. LEU2; rsp5::rsp5-1-K.l. URA3	DKB Lab Collection
Fig 5C and D		
SARY7786	MATa; his3∆1; leu2∆0; met15∆0; ura3∆0; PGPI7::PGAL gpi7::GPI7-9MYC; ted1::kanMX4; dcr2(-255-1490)::LoxP; pRS406-PTPI1- GFP-SNC1-CYC1ter	DKB Lab Collection
SARY8299	MATa; his3∆1; leu2∆0; met15∆0; ura3∆0; PGPI7::PGAL gpi7::GPI7-9MYC; ted1::kanMX4; dcr2(-255-1490)::LoxP; pRS406-PTPI1- GFP-SNC1-CYC1ter; rsp5::rsp5-1-K.l. LEU2	DKB Lab Collection
Fig S1		
SARY5529	MATa; his3∆1; leu2∆0; met15∆0; ura3∆0; gGPI7-9MYC	DKB Lab Collection
SARY5768	MATa; his3∆1; leu2∆0; met15∆0; ura3∆0; PGPI7::PGAL gpi7::GPI7-9MYC	DKB Lab Collection
SARY5769	MATa; his3∆1; leu2∆0; met15∆0; ura3∆0; PGPI7::PGAL gpi7::GPI7-9MYC; ted1::kanMX4; dcr2(-255-1490)::LoxP	DKB Lab Collection
Fig S3A		
SARY5468	MATa; his3∆1; leu2∆0; met15∆0; ura3∆0; sho1::SHO1-EmCitrine-K.l. LEU2	DKB Lab Collection
SARY7536	MATa; his3∆1; leu2∆0; met15∆0; ura3∆0; bst1::KANMX4; sho1::SHO1-EmCitrine-K.l. LEU2	DKB Lab Collection
SARY7538	MATa; his3∆1; leu2∆0; met15∆0; ura3∆0; gup1::KANMX4; sho1::SHO1-EmCitrine-K.l. LEU2	DKB Lab Collection
Fig S3B		
SARY5468	MATa; his3∆1; leu2∆0; met15∆0; ura3∆0; sho1::SHO1-EmCitrine-K.l. LEU2	DKB Lab Collection
SARY5476	MATa; his3∆1; leu2∆0; met15∆0; ura3∆0; gpi7::KANMX4; sho1::SHO1-EmCitrine-K.l. LEU2	DKB Lab Collection
SARY5492	MATa; his3∆1; leu2∆0; met15∆0; ura3∆0; ted1::kanMX4; dcr2(-255-1490)::LoxP; pRS413-gdcr2-6; sho1::SHO1-EmCitrine-K.l. LEU2	DKB Lab Collection
Fig S3C and D		
SARY7782	MATa; his3∆1; leu2∆0; met15∆0; ura3∆0; gGPI7-9MYC; pRS406-PTPI1- GFP-SNC1-CYC1ter	DKB Lab Collection
SARY8525	MATa; his3∆1; leu2∆0; met15∆0; ura3∆0; PGPI7::PGAL gpi7::GPI7-9MYC; pRS406-PTPI1- GFP-SNC1-CYC1ter, ted1::HIS3MX6	DKB Lab Collection
SARY8527	MATa; his3∆1; leu2∆0; met15∆0; ura3∆0; PGPI7::PGAL gpi7::GPI7-9MYC; pRS406-PTPI1- GFP-SNC1-CYC1ter, dcr2::HIS3MX6	DKB Lab Collection
SARY7786	MATa; his3∆1; leu2∆0; met15∆0; ura3∆0; PGPI7::PGAL gpi7::GPI7-9MYC; ted1::kanMX4; dcr2(-255-1490)::LoxP; pRS406-PTPI1- GFP-SNC1-CYC1ter	DKB Lab Collection
Fig S3E		
SARY7817	MATa; his3∆1; leu2∆0; met15∆0; ura3∆0; gGPI7-9MYC; sur7::SUR7-EmCitrine-K.l. LEU2	DKB Lab Collection
SARY7819	MATa; his3∆1; leu2∆0; met15∆0; ura3∆0; PGPI7::PGAL gpi7::GPI7-9MYC; sur7::SUR7-EmCitrine-K.l. LEU2	DKB Lab Collection
SARY7829	MATa; his3∆1; leu2∆0; met15∆0; ura3∆0; PGPI7::PGAL gpi7::GPI7-9MYC; ted1::kanMX4; dcr2(-255-1490)::LoxP; sur7::SUR7-EmCitrine-K.l. LEU2	DKB Lab Collection
Fig S3F		
SARY5897	MATa; his3∆1; leu2∆0; met15∆0; ura3∆0; gGPI7-9MYC; mid2::MID2-EmCitrine-K.l. LEU2	DKB Lab Collection
SARY5899	MATa; his3∆1; leu2∆0; met15∆0; ura3∆0; PGPI7::PGAL gpi7::GPI7-9MYC; mid2::MID2-EmCitrine-K.l. LEU2	DKB Lab Collection
SARY5900	MATa; his3∆1; leu2∆0; met15∆0; ura3∆0; PGPI7::PGAL gpi7::GPI7-9MYC; ted1::kanMX4; dcr2(-255-1490)::LoxP; mid2::MID2-EmCitrine-K.l. LEU2	DKB Lab Collection
Fig S3G and H		
SARY7782	MATa; his3∆1; leu2∆0; met15∆0; ura3∆0; gGPI7-9MYC; pRS406-PTPI1- GFP-SNC1-CYC1ter	DKB Lab Collection
SARY8874	MATa; his3∆1; leu2∆0; met15∆0; ura3∆0; dcw1::KANMX4, dfg5::K.l. LEU2, pRS413-gDCW1, PTPI1- GFP-SNC1-CYC1ter	DKB Lab Collection
SARY8876	MATa; his3∆1; leu2∆0; met15∆0; ura3∆0; dcw1::KANMX4, dfg5::K.l. LEU2, pRS413-gdcw1-3, PTPI1- GFP-SNC1-CYC1ter	DKB Lab Collection
Fig S4A and B		
SARY8312	MATa; his3∆1; leu2∆0; met15∆0; ura3∆0; gGPI7-9MYC; pRS406-gSHO1-EmCitrine	DKB Lab Collection
SARY8314	MATa; his3∆1; leu2∆0; met15∆0; ura3∆0; PGPI7::PGAL gpi7::GPI7-9MYC; pRS406-gSHO1-EmCitrine	DKB Lab Collection
SARY8316	MATa; his3∆1; leu2∆0; met15∆0; ura3∆0; PGPI7::PGAL gpi7::GPI7-9MYC; ted1::kanMX4; dcr2(-255-1490)::LoxP; pRS406-gSHO1-EmCitrine	DKB Lab Collection
Fig S4C and D		
SARY8318	MATa; his3∆1; leu2∆0; met15∆0; ura3∆0; gGPI7-9MYC; pRS406-gSHO1-EmCitrine-DUB	DKB Lab Collection
SARY8320	MATa; his3∆1; leu2∆0; met15∆0; ura3∆0; PGPI7::PGAL gpi7::GPI7-9MYC; pRS406-gSHO1-EmCitrine-DUB	DKB Lab Collection
SARY8322	MATa; his3∆1; leu2∆0; met15∆0; ura3∆0; PGPI7::PGAL gpi7::GPI7-9MYC; ted1::kanMX4; dcr2(-255-1490)::LoxP; pRS406-gSHO1-EmCitrine-DUB	DKB Lab Collection

**Table 2.** Oligo used in this study.

Oligonucleotides	Sequence	Reference or source
FAB1-LoxP-KO-F	TTTCGAATAGCAAGGTAGCTTCCATCCTGTACATGCAAGACCGTCACACAGCcagctgaagcttcgtacgc	DKB Lab Collection
FAB1-LoxP-KO-R	GTGATAGTGTATAAAAAAAAGTTACAGAATATAACTTGTACACGTTTATGTAgcataggccactagtggatctg	DKB Lab Collection
VPS27-LoxP-KO-F	AGATTTTTTTTTGCTAAGGTGAATGAGTAGTGAGTAAAGAACTAAGAACAGTcagctgaagcttcgtacgc	DKB Lab Collection
VPS27-LoxP-KO-R	TATTTATAAGCGCTAGGTTTCTTTTTACAAATACATAGAAAAGGCTACAATAgcataggccactagtggatctg	DKB Lab Collection
DOA4-LoxP-KO-F	TGAGTGTGCACGCTTCCAAAGTTTTTTTTACTATTTGATACATGCTTAAGTTcagctgaagcttcgtacgc	DKB Lab Collection
DOA4-LoxP-KO-R	AACGGGAAAAAAAGTGTATAGACAACGGTTTTCAGTTATTTATTCAAATGAAgcataggccactagtggatctg	DKB Lab Collection
DFG5-LoxP-KO-F	GTTGTTATATAGGACGAACAAATTAGAACGAAATCATATCCAGAACGCAGATcagctgaagcttcgtacgc	DKB Lab Collection
DFG5-LoxP-KO-R	TAGCCTAAATATTAGAATACAATAAAATTTTTTTGAGCCTAGTTTGACACATgcataggccactagtggatctg	DKB Lab Collection

**Table 3.** Reagents and tools.

Reagent/Resource	Reference or source	Identifier or catalog number
Experimental models		
Saccharomyces cerevisiae (BY4741)	DKB Lab Collection	N/A
Strains used in this study	DKB Lab Collection	Table 1
Recombinant DNA		
pRS406-PGALs-mNeon-GAS1	DKB Lab Collection	N/A
pRS406-PGALs-EmCitrine-SED1	DKB Lab Collection	N/A
pRS406-PGALs-SNAPf-GAS1	DKB Lab Collection	N/A
pRS406-PTPI1-GFP-SNC1-CYC1ter	DKB Lab Collection	N/A
pRS406-PTPI1-GFP-snc1-endo-CYC1ter	DKB Lab Collection	N/A
pRS406-PTPI1-DUB-GFP-SNC1-CYC1ter	DKB Lab Collection	N/A
pRS406-PTPI1-dub*-GFP-SNC1-CYC1ter	DKB Lab Collection	N/A
Antibodies		
Rabbit polyclonal anti-Gas1p	Kindly provided by Prof. Howard Reizman (University of Geneva)	N/A
Mouse monoclonal anti-ALP	Invitrogen	Cat#A6458
Mouse monoclonal anti-Pma1p	Invitrogen	Cat#MA1-91567
Mouse monoclonal anti-Pgk1p	Molecular Probes	Cat#459250
Rabbit polyclonal anti-GFP	Sigma-Aldrich	Cat#G1544
Mouse monoclonal anti-MYC	Roche	Cat#11667149001
Mouse monoclonal anti-Dpm1	Invitrogen	Cat#A-6429
Donkey anti-rabbit IgG	GE Healthcare	Cat#NA934
Goat anti-mouse IgG	Sigma-Aldrich	Cat#A8924
Oligonucleotides and other sequence-based reagents		
PCR primers	This study	Table 2
Chemicals, enzymes, and other reagents		
Galactose	Sigma-Aldrich	Cat#G0750
Raffinose	Sigma-Aldrich	Cat#R0250
LIVE/DEAD Yeast Viability Kit	Molecular Probe	Cat#L7009
Acid-washed glass beads	Sigma-Aldrich	Cat#G8772
Pefabloc SC	Roche	Cat#11429876001
EDTA-free protease inhibitor cocktail	Roche	Cat#11873580001
Triton X-100	Sigma-Aldrich	Cat#X100
OptiPrep Density Gradient Medium	Sigma-Aldrich	Cat#D1556
Trichloroacetic acid	Sigma-Aldrich	Cat#T8657
Di-4-ANEPPDHQ	Invitrogen	Cat#D36802
FM 4-64	Invitrogen	Cat#T13320
SNAP-Surface 488	New England Biolabs	Cat#S9124S
Concanavalin A	Sigma-Aldrich	Cat#C7275
Software		
Fiji	https://fiji.sc	
Adobe Photoshop 2024	https://www.adobe.com	
Adobe Illustrator 2024	https://www.adobe.com	
Prism 10	https://www.graphpad.com	
Microsoft Office 365	https://www.microsoft.com	
Other		
LSM 980	Zeiss	

### Experimental details

#### Quantification of the SNAPf-Gas1p fluorescence intensity signal on the PM

The region of interest was identified using the morphological segmentation and the morphological filter functions with the ImageJ plugin MorphoLibJ (Legland et al, 2016). Fluorescence intensity from the region of interest was measured from six cells, and the mean fluorescent signal intensity was determined for the mother [mean _mother_] and daughter [mean _daughter_] cells. The ratio of the mean intensity (daughter versus mother) was obtained using the equation: ratio = [mean _daughter_]/[mean _mother_] (Fig S2A). The percentage of SNAPf-Gas1p on the plasma on cells from various yeast strains compared with the WT cells was calculated using this equation: percentage = [mean _strain_]/[mean _WT_], where [mean] = [mean _mother_] + [mean _daughter_] (Fig S2B) is calculated using the mean values calculated for the ratio of mean intensity (Fig S2A).

### Induction of Gpi7p synthesis

Induction of Gpi7p synthesis was conducted essentially as described in Chen and Banfield (2022). In brief, yeast strains were cultured in YEPD at 25°C overnight and thereafter diluted in YEPD to obtain a cell density of 0.3 × 10^7^ cells/ml and grown for a further 3 h at 25°C. Cells were collected by centrifugation and washed once with YEP (i.e., in the absence of glucose), resuspended in YEPGR containing 2% galactose (Cat#G0750; Sigma-Aldrich) and 1% raffinose (Cat#R0250; Sigma-Aldrich), and incubated at 25°C for 4 h to induce the expression of Gpi7p. Depending on the experimental design (see the main text for details), cells were either cultured in YEPGR for an additional 2 h (to achieve an ideal fluorescent signal intensity of GPI-AP markers) or washed with YEP, resuspended in YEPD, and incubated for an additional 3 h at 25°C, to minimize the influence of Gpi7p overexpression and to recover the cells from a less preferable carbon source.

### Yeast cell viability test

1 × 10^7^ yeast cells were collected by centrifugation and then washed once with buffer (10 mM Hepes, pH 7.2, containing 2% glucose). Cell pellets were resuspended in 1 ml of wash buffer to which 1 μl of Component A from the LIVE/DEAD Yeast Viability Kit (Cat#L7009; Molecular Probe) and 5 μl of Component B were added. Cells suspended in the viability assay were incubated at 25°C in the dark for 30 min, washed once with wash buffer, and thereafter viewed by fluorescence microscopy (as per the manufacturer’s instructions). The percentage of viable cells was calculated as follows: percentage of cell viability = (total # cells–total # dead cells)/total # cells x100.

### DRM isolation

The DRM isolation procedure was performed based on the method described by Bagnat et al (2000). In brief, 5 × 10^8^ cells were collected from log phase cultures and washed once with TNE buffer (50 mM Tris–HCl, pH 7.4, 150 mM NaCl, 5 mM EDTA) before being stored at −80°C. Cells were thawed on ice and thereafter lysed at 4°C using acid-washed glass beads (Cat#G8772; Sigma-Aldrich) in 1 ml TNE buffer containing a protease inhibitor mixture 1 mM Pefabloc SC (Cat#11429876001; Roche) and 1×EDTA-free protease inhibitor cocktail (Cat#11873580001; Roche). Unbroken cells were removed by two rounds of centrifugation at 500*g* for 5 min. The resulting lysate was adjusted to a final concentration of 1% Triton X-100 (Cat#X100; Sigma-Aldrich) and thereafter incubated on ice for 30 min. A total of 250 μl Triton X-100–treated lysate was mixed with 500 μl OptiPrep Density Gradient Medium (Cat#D1556; Sigma-Aldrich) containing protease inhibitors generating a final 40% iodixanol mixture. Lysate (628 μl) was loaded onto the bottom of an ultracentrifugation tube (for a S55-S rotor; Thermo Fisher Scientific) and overlaid with 1,005 μl of 50% OptiPrep medium in TXNE buffer (TNE buffer containing 0.1% Triton X-100 and protease inhibitors). An additional 167 μl of TXNE buffer/protease inhibitor mixture was layered onto the top of the sample bringing the final volume to 1,800 μl. Samples were centrifuged at 55,000*g* for 2 h at 4°C in a S55-S rotor. After centrifugation, six 300 μl fractions were collected from the top of the centrifugation tube. Proteins in each 300 μl fraction were precipitated by the addition of 600 μl ice-cold 15% trichloroacetic acid (TCA) (Cat#T8657; Sigma-Aldrich), and after mixing, each fraction was incubated at −20°C overnight. Precipitated proteins were collected by centrifugation in an Eppendorf microcentrifuge at 4°C and 16,100*g* for 10 min. Sedimented proteins were washed once with cold acetone (−20°C) and thereafter dissolved in SDS–PAGE sample buffer, heated to 95°C for 5 min after which protein samples were subject to SDS–PAGE and immunoblotting.

### Lipid ordered/disordered imaging and quantification

Lipid ordered/disordered imaging and quantification was performed essentially as described by Owen et al (2011). In brief, a total of 10^7^ yeast cells were cultured in YEPD or in YEPGR for 4 h and thereafter in YEPD for an additional 3 h. Yeast cells were collected by centrifugation, washed once with ice-cold YEPD, and then resuspended in 1 ml ice-cold YEPD containing 5 μM di-4-ANEPPDHQ (Cat#D36802; Invitrogen). The cell di-4-ANEPPDHQ suspensions were incubated on ice in the dark for 20 min, after which the stained cells were washed three times with ice-cold PBS, mounted onto slides, and viewed with a Leica SP8 confocal microscope.

A wavelength of 488 nm was used to excite di-4-ANEPPDHQ, and the emission signal was collected between 500–580 and 620–750 nm to acquire images corresponding to ordered and disordered lipids, respectively. The generalized polarization (GP) value was calculated using the following equation: GP=(I_500–580_ − GI_620–750_)/(I_500-580_ + GI_620-750_). The G factor was calculated using the equation: G=(GP_ref_ + GP_ref_GP_mes_ − GP_mes_ − 1)/(GP_mes_ + GP_ref_GP_mes_ − GP_ref_ − 1). The value of GP_ref_ was −0.85 for di-4-ANEPPDHQ, whereas GP_mes_ was the GP value for a 5 mM solution of a di-4-ANEPPDHQ dye in DMSO.

The GP value and the hue–saturation–brightness images were generated using the ImageJ macro provided by Owen et al (2011). Regions of interest were selected manually by adjusting the threshold of the grayscale image in ImageJ. The moving average trendline of the GP value histogram (Fig 1I) was added using Excel (Microsoft).

### Labeling vacuoles with an FM 4-64 dye

10^7^ yeast cells were cultured in YEPD or YEPGalRaf for 4 h, collected by centrifugation, and resuspended in 50 μl of YEPD containing 30 μM FM 4-64 (Cat#T13320; Invitrogen). Yeast cells were incubated at 25°C for 30 min, harvested by centrifugation, and washed once with YEPD and thereafter incubated in 2 ml of YEPD at 25°C for 120 min before being subject to epifluorescence microscopy. The Z-stack images of FM 4-64–labeled vacuoles from IPEM2-GR cells were obtained using confocal microscopy with Zeiss LSM 980.

### Monitoring endocytosis with FM 4-64 and SNAP-Surface 488 dyes

Cultured yeast cells were harvested by centrifugation and resuspended in ice-cold PBS on ice for 10 min. Chilled cells were thereafter resuspended in ice-cold PBS containing 30 μM FM 4-64 (Cat#T13320; Invitrogen) and 5 μM SNAP-Surface 488 (Cat#S9124S; NEB), and incubated on ice for 30 min. After incubation with FM 4-64 and SNAP-Surface 488, cells were washed twice with ice-cold PBS to remove excess dyes. Yeast cells were either immediately mounted onto concanavalin A (Cat#C7275; Sigma-Aldrich)–coated slides for microscopy or cultured in YEPD medium for 2 h (to monitor the intracellular trafficking of FM 4-64 and SNAPf-Gas1p) before being subject to confocal microscopy.

### Monitoring endocytosis of IPEM2-GR and Vph1p-mNeon-labeled WT cells with an FM 4-64 dye

IPEM2-GR and WT cells were grown under identical conditions and collected by centrifugation. Equal numbers of cells from both strains were mixed together and resuspended in ice-cold PBS on ice for 10 min. Chilled cells were thereafter resuspended in ice-cold PBS containing 30 μM FM 4-64 (Cat#T13320; Invitrogen) and incubated on ice for a further 30 min. After incubation with FM 4-64, cells were washed twice with ice-cold PBS to remove excess dyes. Yeast cells were immediately mounted onto a concanavalin A (Cat#C7275; Sigma-Aldrich)–coated 35-mm confocal dish (SPL, Cat#100350) and covered with 2 ml of complete synthetic defined medium containing 2% glucose. Endocytosis was monitored, and images were captured over the course of 160 min by confocal microscopy with Zeiss LSM 980.

### Epifluorescence microscopy

After yeast strain incubation, cells were washed and resuspended in PBS. Aliquots of yeast cell suspensions (0.8 μl) were placed onto slides coated with concanavalin A (Cat#7275; Sigma-Aldrich) and examined by epifluorescence microscopy. Cells were photographed immediately after examination.

Cell visualization and photography were performed using a Nikon ECLIPSE 80i microscope (Nikon Instruments) equipped with a Nikon Plan Apo VC 100X/1.40 oil objective lens and a SPOT-RT3 monochrome camera (Diagnostic Instruments, Inc.). Images were acquired using SPOT software (version 4.6; Diagnostic Instruments, Inc.). Digital images were processed using Photoshop CS6 software (Adobe Systems).

### Sample preparation for immunoblotting

10^7^ yeast cells were collected by centrifugation and thereafter resuspended in 15% TCA (Cat#T8657; Sigma-Aldrich). The cell–TCA suspension was stored at −20°C overnight after which the resulting precipitate was collected by centrifugation and washed once with cold acetone. After being air-dried, the precipitate was dissolved in SDS–PAGE sample buffer containing 50 mM NaOH and 0.5% SDS. The sample was then subject to SDS–PAGE and transferred to a nitrocellulose membrane overnight using a current of 250 mA.

### Immunoblotting

Where the molecular weights of proteins of interest were too similar to permit simultaneous immunoblotting, by cutting membranes to separate the proteins of interest, the same protein samples were resolved on separate gels, transferred to nitrocellulose membranes, and immunoblotted with the desired antibodies and an antibody against Pgk1p (which served as a gel loading control; please see Table 3 for further details).

### Quantification of immunoblots

For Fig 1E, the ratio (mGas1p/pGas1p) from each fraction in Fig 1C and D was calculated using the formula: Ratio = I_m_/I_p_. Here, “I_m_” represents the signal intensity of mNeon-mGas1p in each fraction, and “I_p_” represents the signal intensity of mNeon-pGas1p in each fraction.

The percentage of mNeon-mGas1p from each fraction in Fig 1C and D was calculated using the formula: Percentage = I_n_/I_total_ (x100). In this formula, “I_n_” represents the signal intensity of mNeon-mGas1p in each fraction, whereas “I_total_” is the sum of the signal intensity of mNeon-mGas1p from all six fractions (Fig 1F).

To calculate the ratio (s-ALP/m-ALP), the following formula was used: Ratio = I_s_/I_m_. Here, “I_s_” represents the signal intensity of s-ALP in each fraction (fractions 4–6), and “I_m_” represents the signal intensity of m-ALP in each fraction (fractions 4–6) (Fig 1G).

The ratio (in arbitrary units, a.u.) of free GFP (Figs 3B and 4C, E, and F) was calculated using the following formula: Ratio = I_GFP_/I_GFP-Snc1p_. In these formulas, “I_GFP_” refers to the signal intensity of free GFP in each sample, and “I_GFP-Snc1p_” refers to the signal intensity of GFP-Snc1p in the same sample.

### Quantification of protein colocalization

The calculation of Pearson’s coefficient was performed using the JACoP ImageJ plugin (Bolte & Cordelières, 2006). The regions of cells of interest were cropped and subject to JACoP, and Costes’ automatic threshold was applied to the paired cropped images to calculate Pearson’s coefficient.

## Supplementary Material

Reviewer comments
